# Potential field mechanisms and distributed learning for enhancing the navigation of micro/nanorobot in biomedical environments

**DOI:** 10.1016/j.heliyon.2024.e35328

**Published:** 2024-07-26

**Authors:** Junqiao Zhang, Qiang Qu, Xuebo Chen

**Affiliations:** School of Electronics and Information Engineering, University of Science and Technology Liaoning, Anshan, 114051, China

**Keywords:** Complex system, Collective behavior, Potential fields, Micro/nanorobots, Distributed learning, Local interactions

## Abstract

In complex systems, single micro/nanorobots encounter challenges related to limited loading capacity and navigation, hindering their effective utilization in targeted therapy and drug delivery. To solve these challenges, this paper explores potential field mechanisms as a means to simulate natural collective behavior. This approach aims to enhance the navigation and efficiency of micro/nanorobots in high-demand therapeutic areas. The mechanism enables micro/nanorobots to dynamically adapt to environmental gradients, minimizing off-target effects while maximizing therapeutic efficacy and enhancing robustness through redundancy. Additionally, this study introduces innovative distributed learning and cooperative control strategies. Each micro/nanorobot updates its navigation strategy through local interactions and influences with the dynamic environment. This allows micro/nanorobots to share information and improve their navigation toward therapeutic targets. The simulation results demonstrate that collective behavior and potential field mechanisms can enhance the precision and efficiency of targeted therapy and drug delivery in dynamically changing environments. In conclusion, the proposed approach can improve the limitations of single micro/nanobot, offering new possibilities for the development of advanced therapeutics and drug delivery systems.

## 1 Introduction

Collective behavior is an interdisciplinary field that integrates insights from computer science, medicine, biology, and physics, reflecting the complexity and diversity of the field [[1], [2], [3]]. This interdisciplinarity mainly originates from simple local interactions between individuals, whether biological organisms (such as cells, flocks of birds, schools of fish) or artificial agents (such as groups of nanorobots) [4,5]. The phenomenon of collective behavior, from the synchronized movements of biological organisms to the coordinated actions of micro/nanorobots, exhibits the emergent nature of complex systems [6,7]. These systems demonstrate adaptability and precise coordination of actions to dynamic environments, thus requiring a modeling approach that can adapt to such conditions [8,9]. Especially in the field of targeted therapeutics and drug delivery, understanding and exploring the collective behavior of micro/nanorobots becomes crucial.

The emergence of micro/nanorobots means a new era in the field of targeted therapy and precision drug delivery, offering innovative and interdisciplinary approaches to tackle a variety of health challenges [10,11]. Single micro/nanorobot, despite their innovative applications in targeted therapy and drug delivery, inherently possess limitations that can be significantly amplified in complex biomedical environments [12]. Firstly, one of the major limitations of single micro/nanorobot is their constrained loading capacity [13]. Due to their small size, these robots can only transport a limited number of therapeutic drugs. This limitation requires the deployment of a large number of robots, which can complicate the delivery system and increase the risk of loading, or require multiple doses to achieve the desired therapeutic effect [14]. The latter can lead to reduced patient compliance and increased treatment costs, posing significant challenges in clinical applications. Secondly, the complex and dynamic nature of the human body poses significant navigational challenges for single micro/nanorobots [15]. Factors such as unpredictable fluid flows, variable tissue densities, and intricate biochemical signals can severely impede a robot's ability to efficiently reach targeted sites [16]. Without the ability for real-time adaptation or the support of a collective intelligence, the chances of a single robot successfully navigating to its destination are considerably reduced [17]. Furthermore, the efficiency and precision of drug delivery can also be affected when relying on single micro/nanorobots [18]. This can lead to the wastage of therapeutic agents and reduced treatment efficacy, as well as an increased risk of side effects due to off-target activity. Finally, the effectiveness of single micro/nanorobots is vulnerable to the body's immune response and other environmental factors. Isolated robots face a higher risk of being recognized and eliminated by the immune system or becoming ensnared in biological barriers, which prevents them from delivering their therapeutic payload to the intended site [19].

The potential field mechanism provides a powerful solution to these challenges by simulating collective behavior in natural systems. The concept of potential fields plays a key role in collective behavior, representing the peak of the therapeutic target and attracting micro/nanorobots to navigate toward the areas most in need of treatment [20]. The closer the robots are to the source of the potential field, the stronger the attraction or repulsion is felt, which is crucial for controlling the collective behavior of the robots, especially for group formation and navigation [21]. Through the principle of potential field, micro/nanorobots can form collective behaviors in the biomedical environment, identify and navigate to the optimal location for treatment and drug delivery [22]. In addition, the concept of potential field is also used to simulate complex environmental dynamics in the field of artificial intelligence, such as urban crowd movement or animal migration, describing the movement and decision-making processes of individuals or groups [23,24]. The potential field, as a dynamic mathematical model, depicts the spatial distribution of attractive and repulsive forces generated by the therapeutic target, while taking into account environmental factors such as temperature and chemical concentration [25]. Specifically, the core of this approach is to enable micro/nanorobots to recognize and move toward potential field peaks that represent the highest therapeutic needs [26]. This means that the micro/nanorobots are designed to autonomously adjust their action paths in response to environmental gradients in the vicinity of the therapeutic target, such as temperature or chemical concentrations [27,28]. In this way, they are able to deliver drugs directly to the lesion site, maximizing the therapeutic effect while avoiding unnecessary damage to the surrounding healthy tissue [22,29]. The application of this technique embodies the characteristics of adaptive and decentralized decision-making.

By imitating these properties, micro/nanorobots can behave more flexiblely and efficiently, and be able to adapt to complex and variable biomedical environments [30]. This is not only a way to model the collective behavior of organisms in nature, but also a common approach in the design of robots and autonomous systems that aim to achieve efficient, targeted therapy by mimicking the dynamics of nature [31]. This dynamic mathematical model enables micro/nanorobots to operate as a coordinated swarm, enhancing their capabilities beyond the limitations of individual operation. In a potential field environment, each point in space is associated with a potential value, which micro/nanorobots use to navigate by moving from lower to higher potential values. This mechanism allows for the efficient, targeted navigation of micro/nanorobots towards areas of high therapeutic need by interpreting environmental gradients as cues for movement [32]. When applied to a swarm of micro/nanorobots, the potential field model exponentially increases the collective efficacy of the swarm [33]. Each micro/nanorobot is influenced by local interactions and potential fields, contributes to achieve emergent behavior of the swarm and perform complex tasks that would be impossible for a single micro/nanorobot [34].

Consequently, the relationship between the response of the micro/nanorobots to the potential fields and the intelligence of micro/nanorobots could be further discussed. Firstly, the ability of micro/nanorobots to respond to potential fields is directly linked to their level of artificial intelligence. Micro/nanorobots with advanced AI capabilities can recognize and prioritize specific potential fields, which guide them toward therapeutic targets with greater precision. This targeted approach minimizes side effects and maximizes therapeutic efficacy, which is particularly important in treating diseases with dispersed or multifocal lesions. By incorporating AI algorithms, these robots can continuously learn from their environment, improving their navigation strategies and therapeutic outcomes over time. Secondly, intelligent micro/nanorobots can process gradient information from potential fields to make autonomous decisions about their movement. This involves moving towards regions with higher potential values (therapeutic targets) and away from regions with lower potential values or obstacles. The capability to make such decisions in real time without external control signifies a form of distributed intelligence, where each robot independently contributes to the collective goal. These robots can communicate and coordinate with each other, much like a swarm, to achieve complex tasks that are beyond the capabilities of single units. The swarm can distribute itself optimally across a target area, ensuring efficient drug delivery and minimizing off-target effects. In addition, the response to potential fields also contributes to the robustness of micro/nanorobot swarms. In a biomedical context, individual robots might encounter various challenges such as immune system attacks or physical barriers. However, the collective response guided by potential fields ensures that even if some robots fail, others can continue the task. This redundancy is a hallmark of intelligent systems, providing reliability and continuity in therapeutic interventions. Finally, intelligent design ensures that these robots can learn from their interactions and refine their responses to new potential fields. Their ability to learn, adapt, and coordinate ensures that they can navigate through the body's intricate environments, delivering targeted therapies with minimal side effects. This enhances the overall therapeutic efficacy and opens new possibilities for treating various medical conditions.

In this study, we explore the use of innovative distributed learning and cooperative control strategies to drive the collective behavior of micro/nanorobots in the field of targeted therapy and drug delivery. Compared with traditional control methods, our strategy enables micro/nanorobots to independently collect data within limited resources and communication ranges, acquiring information through local interactions within a swarm. This collective information sharing mechanism mimics the resource seeking behavior in nature [35], such as a shoal of fish moving collectively to resource-rich or more efficient treatment areas through a gradient lifting algorithm, allowing each micro/nanorobot to continuously improve its treatment target search strategy [36]. Our simulations validate the effectiveness of this approach in an uncertain and dynamically changing environment, particularly in its ability to rapidly collaborate to identify therapeutic targets. Compared to the lack of interaction and communication of a single robot, our strategy improves the speed and efficiency of task execution, emphasizing the importance of distributed information sharing and collective action in achieving group goals. The research emphasizes potential field mechanisms and collective behavior to improve the precision and efficiency of micro/nanorobots in targeted medicine. Finally, we reveal the complex relationship between individual behavior and collective behavior, providing valuable insights for the development of more complex targeted therapies and drug delivery systems.

The rest of the paper is structured as follows: Section 2 provides our conceptual framework, focusing on collective behavior and the mechanisms of potential fields. In Section 3 introduces distributed learning and cooperative control strategies. Section 4 present the convergence analysis. In Section 5, we discuss the simulation results. Finally, Section 6 and Section 7 summarizes our findings and future research, respectively.

## 2 Mobile sensing individual network

### 2.1 Models for mobile perception individual

We consider the dynamics of individual behavior and local interaction within a monitoring area $M\subset R^{2}$, which is a convex compact set. Let *n* denotes the number of individuals randomly distributed on the monitoring region $M\subset R^{2}$. $l=\{1,2,.\;.\;.,N_{s}\;\}$ index the identity of each individual [37]. At time *t*, $q_{i}\left(t\right)\in M$ is defined as the location of the *i*th individual and $q=col\left(\;q_{1},q_{2},.\;.\;.,q_{N_{s}\;}\right)\in R^{2Ns}$ is the configuration of the complex system. The discrete-time high-order dynamic model is expressed as follows:

(1) $$\left\{ \begin{array}{c} q_{i}\left(t+\Delta_{t}\right)=q_{i}\left(t\right)+\Delta_{t}p_{i}\left(t\right)\\ p_{i}\left(t+\Delta_{t}\right)=p_{i}\left(t\right)+\Delta_{t}u_{i}\left(t\right) \end{array}\right.$$

where $q_{i}$, $p_{i}$ and $u_{i}$ are the location, velocity and the control input of individual *i* at time $t\in R_{\geq 0}$, respectively. Δt denotes the sampling time or iteration step size. Let the measured value of the *i*th individual at its position $q_{i}\left(t\right)$ and sampling time t be $y\left(q_{i}\left(t\right)\right)$, including the values of the potential field $F\left(q_{i}\left(t\right)\right)$ and the individual noise w(t):

(2) $$y\left(q_{i}\left(t\right)\right)=F\left(q_{i}\left(t\right)\right)+w\left(t\right)$$

where $F:M\to \left[0,\mu_{\max}\right]$ is the peak of the potential field.

### 2.2 Potential field model

Considering the collective motion of *n* individuals in *m* -dimensional space, we study a set of motion control problems for multiple individuals in pursuit of the potential field. Each individual has the ability to perceive and search for positions in the potential field, including sensing disease-related biomarkers, understanding cellular signals, or detecting changes within tissues. Subsequently, the information collected by the individuals is used to locate and search the potential field [38]. Therefore, the potential field model is defined as follows:

(3) $$F=\Theta \Phi^{T}=\sum_{j=1}^{K}\theta_{j}\phi_{j}$$

where $\Theta =\left[\theta_{1},\theta_{2},...,\theta_{k}\right]$, and $\Phi^{T}=\left[\phi_{1},\phi_{2},...,\phi_{j}\right]$, *j* and *K* denote the index and the total number of function distributions, respectively. Θ and $\Phi^{T}$ are presented respectively as:

(4) $$\begin{array}{c} \Theta ={\left[\begin{array}{cccc} \theta_{1} & \theta_{2} & \cdots & \theta_{k} \end{array}\right]}^{T}\in \Theta \\ \Phi^{T}=\left[\begin{array}{cccc} \phi_{1} & \phi_{2} & \cdots & \phi_{k} \end{array}\right] \end{array}$$

where $\theta \subset R^{m}$ is a compact set. $\left\{\phi_{j}\left(p\right)\right\}$ is Gaussian radial basis functions and given by:

(5) $$\phi_{i}\left(p\right)=\frac{1}{\beta_{j}}\exp\left(\frac{-{\left\|p-\zeta_{j}\right\|}^{2}}{\sigma_{j}^{2}}\right),\forall j\in M\text{,}$$

where M={1,…,m}, $\beta_{j}$ serves as the normalizing constant and $\sigma_{j}$ represents the Gaussian basis width. The basis functions are centered at points $\{\xi_{j}\;|\;j\in \;M\}$, uniformly distributed within the monitored region *Q*. The partial derivative of $\phi \left(x\right)\in R_{m\times 1}$ with respect to $x\in R_{2\times 1}$ at the specific point $x^{*}$ is defined as $\phi^{\prime }\left(x^{*}\right)$ and is presented as follows:

(6) $$\phi^{\prime }\left(v^{*}\right)={\frac{\partial \phi \left(x\right)}{\partial x}|}_{v=v^{*}}\in R^{m\times 2}$$

The gradient of the potential field at $q_{i}$ can be defined as:

(7) $$\nabla F\left(q_{i}\right)={\frac{\partial \phi^{T}\left(x\right)}{\partial x}|}_{x=q_{i}}\in R^{2\times 1}$$

By utilizing Eq. (3), Eq. (7) can be expressed using *θ*.

(8) $$\nabla F\left(q_{i}\right)={\frac{\partial \phi^{T}\left(x\right)}{\partial x}|}_{x=q_{i}}\Theta =\phi^{\prime T}\left(q_{i}\right)\Theta \in R^{2\times 1}$$

The estimate of $\nabla F\left(q_{i}\right)$ based on $\hat{\theta }$ is represented as $\nabla \hat{F}\left(q_{i}\right)$. Now, we consider the movement of *n* individuals in a two-dimensional space. We model the potential field in a 2-D space of (*x*, *y*) as:

(9) $$F\left(x,y\right)=\Theta \Phi^{T}\left(x,y\right)=\sum_{j=1}^{K}\theta_{j}\phi_{j}\left(x,y\right)$$

where *Θ* = [*θ*_1_, *θ*_2_, …,*θ*_*K*_], and $\Phi \left(x,y\right)=\left[\phi_{1},\phi_{2},...,\phi_{K}\right]$, *ϕ*_*j*_ (*x*, *y*) is a function of the density distribution, and *θ*_*j*_ denotes the weight of the density distribution of the function *ϕ*_*j*_ (*x*, *y*). *j* and *K* is the index and total number of density distribution functions, respectively [39]. In addition, this study extends existing potential field mechanisms to include intrinsic properties causing attraction and repulsion among micro/nanorobots, aiming to enhance their collective behavior and navigation efficiency. Thus, introduce an additional term in the potential function to account for the intrinsic properties that cause attraction or repulsion among the micro/nanorobots. This term can be represented as $U_{\mathrm{int}}\left(r_{ij}\right)\text{,}$ where $r_{ij}$ is the distance between micro/nanorobot *i* and *j*. The total potential function $U_{\text{total}}\left(q\right)$ for a micro/nanorobot can be represented as:

(10) $$U_{total}\left(q_{i}\right)=U_{field}\left(q_{i}\right)+\sum_{j\neq i}U_{\mathrm{int}}\left(r_{ij}\right)$$

where $U_{\text{field}}\left(q_{i}\right)$ is the potential field due to external factors (e.g., therapeutic target), and $U_{\mathrm{int}}\left(r_{ij}\right)$ represents the intrinsic potential due to attraction or repulsion among the micro/nanorobots. Then, the intrinsic potential function $U_{\mathrm{int}}\left(r_{ij}\right)$ can be defined as:

(11) $$U_{\mathrm{int}}\left(r_{ij}\right)=\left\{ \begin{array}{c} \alpha \;\log\left(r_{ij}/d\right)\;if\;r_{ij}<d\\ \beta /r\;if\;r_{ij}\geq d \end{array}\right.$$

where α and β are constants, and *d* is a threshold distance determining when attraction turns into repulsion. By incorporating these intrinsic attraction and repulsion forces into the potential field model, the proposed approach aims to enhance the navigation and collective behavior of micro/nanorobots in complex biomedical environments.

### 2.3 Collective behavior

In biomedical environments, each individual needs to bypass obstacles, which is controlled by the artificial potential energy function [40]. In addition, a smooth collective potential function is introduced [41], where it is necessary for individuals to follow a set of algebraic constraints as defined by $\left\|q_{i}-q_{j}\right\|=d$ for all j∈N(i,q).

(12) $$U_{1}\left(q\right)=\sum_{i}\sum_{j\in N\left(i,q\right),j\neq i}U_{ij}\left(q_{i}-{q_{j}}^{2}\right)=\sum_{i}\sum_{j\in N\left(i,q\right),j\neq i}U_{ij}\left(r_{ij}\right)$$

where $r_{ij}={\left\|q_{i}-q_{j}\right\|}^{2}$. The attractive/repulsive potential function $U_{ij}\left(r_{ij}\right)$ is represented as:

(13) $$U_{ij}\left(r_{ij}\right)=\frac{1}{2}\left(\log\left(\alpha +r_{ij}\right)+\frac{\alpha +d^{2}}{\alpha +r_{ij}}\right),if\;r_{ij}<d_{0}^{2}\text{,}$$

otherwise ($i.e.,rij\geq d_{0}^{2}$), it is defined based on the gradient of the potential function. Where $\alpha ,d\in R_{>0}$ and $d\;<\;d_{0}$. The gradient of the potential functions of individual *i* relative to *q*_*i*_ is defined as:

(14) $$\nabla U_{1}\left(q_{i}\right)=\frac{\partial U_{1}\left(q\right)}{\partial q_{i}}={\sum_{j\neq i}\frac{\partial U_{ij}\left(r\right)}{\partial r}|}_{r=r_{ij}}2\left(q_{i}-q_{j}\right)=\left\{ \begin{array}{c} \begin{array}{cc} \sum_{j\neq i}\frac{\left(r_{ij}-d^{2}\right)\left(q_{i}-q_{j}\right)}{{\left(\alpha +r_{ij}\right)}^{2}} & if\;r_{ij}<d_{0}^{2} \end{array}\\ \begin{array}{cc} \sum_{j\neq i}\rho \left(\frac{\sqrt{r_{ij}}-d_{0}}{\left|d_{1}-d_{0}\right|}\right)\frac{d_{0}^{2}-d^{2}}{{\left(\alpha +d_{0}^{2}\right)}^{2}}\left(q_{i}-q_{j}\right) & if\;r_{ij}\geq d_{0}^{2} \end{array} \end{array}\right.$$

where ρ: R ≥ 0→[0, 1] is the bump function. In Eq.s (12), (13), (14), a non-zero gain factor α is introduced to regulate the reaction force between individuals. The potential *U*_2_ can avoid collisions between individuals. The total potential function is defined as:

(15) $$U\left(q\right)=k_{1}U_{1}\left(q\right)+k_{2}U_{2}\left(q\right)$$

where $k_{1},k_{2}\in R_{≧0}$ are weighting factors. Individuals use cooperative strategies to seeking the peak of the potential field. This method simulates the process of individuals collecting and sharing information within constraints relevant to targeted therapy and drug delivery.

Section 2.3 explains the basic principles of collective behavior in micro/nanorobots, emphasizing the use of artificial potential energy functions to facilitate obstacle avoidance and structured navigation. These principles ensure that robots maintain a certain distance from each other, prevent collisions, and preserve group formation. Building on these concepts, Section 3 introduces the application of distributed learning and cooperative control to achieve effective navigation and collective behavior. In distributed learning, local data collection and information sharing with neighboring robots enable each robot to dynamically estimate potential field peaks. This iterative process allows the swarm to continuously refine its understanding of the environment, enhancing navigation towards therapeutic targets. Cooperative control strategies further utilize the estimated potential fields and information from neighboring robots to enhance navigation efficiency. By integrating these strategies with the foundational principles from Section 2.3, the robots can maintain safe distances and stable structures while dynamically adapting to changing environments. This synthesis of collective behavior principles with distributed learning and cooperative control demonstrates a coherent approach to improving the precision and efficiency of micro/nanorobot navigation in targeted therapy and drug delivery applications.

## 3 Distributed learning and cooperative control

**Fig. 1:**
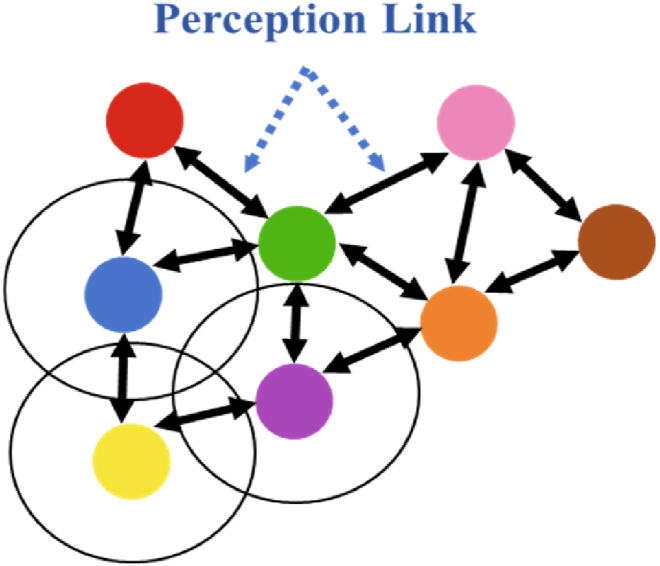
Perceptual links between individuals. Each colored circle represents an individual, the black circle represents the perceptual radius, and the black line represents the interaction between individuals.

Distributed learning allows a single micro/nanobot to gather information from its surroundings and communicate with nearby peers, as shown in Fig. 1. This decentralized approach enables the swarm to dynamically adapt to the environment, optimizing their collective behavior. By continuously exchanging information, the micro/nanorobots can update their internal states and decisions based on the latest data, leading to a more robust and flexible system. Then, cooperative control algorithms guide each micro/nanorobot to navigate towards the peak of the potential field, ensuring precise targeted therapy and efficient drug delivery. This control algorithms ensure that the swarm works cohesively, with each robot adjusting its path to avoid obstacles and maintain optimal formation. By using distributed learning and cooperative control, the swarm can enhance its navigation capabilities and achieve higher accuracy in biomedical applications.

### 3.1 Distributed learning

In the application of micro/nanorobot swarms within biomedical environments, the effective navigation of a single micro/nanorobot is crucial. This navigation is often guided by potential fields, which can represent various gradients such as chemical concentrations, temperature variations, or other relevant biomedical signals. Each micro/nanorobot within the swarm uses a distributed learning algorithm to estimate the peak of the potential field F:M→[0,F:max]. Let F(p) be the potential field is described as:

(16) $$F\left(p\right)=\sum_{j=1}^{k}\phi_{j}\left(p\right)\theta_{j}=\phi^{T}\left(p\right)\Theta$$

where $\phi^{T}\left(p\right)$ and θ are presented respectively as:

$$\begin{array}{c} \phi^{T}\left(p\right)=\left[\begin{array}{cccc} \phi_{1}\left(p\right) & \phi_{2}\left(p\right) & \cdots & \phi_{k}\left(p\right) \end{array}\right]\text{,}\\ \Theta ={\left[\begin{array}{cccc} \theta_{1} & \theta_{2} & \cdots & \theta_{k} \end{array}\right]}^{T}\in \Theta \end{array}$$

where $\theta \subset R^{m}$ is a compact set. $\left\{\phi_{j}\left(p\right)\right\}$ is Gaussian radial basis functions and given by:

(17) $$\phi_{j}\left(p\right)=\frac{1}{\beta_{j}}\exp\left(\frac{-{\left\|s-p_{j}^{c}\right\|}^{2}}{\sigma_{j}^{2}}\right)$$

where $\beta_{j}$ is a normalizing constant and $\sigma_{j}$ denotes the width of the Gaussian basis. Centers of basis functions $p_{j}^{c}$ for j∈{1,…,k} are evenly distributed in the monitoring area *R*. $\Theta \in \Theta \subset R^{m}$ is the true parameter in Eq. (16). Subsequently, in Eq. (2), the micro/nanorobots is observed at the position $p_{k}$,

(18) $${y\left(p_{k}\right)=\phi }^{t}\left(p_{k}\right)\Theta +w\left(k\right)$$

where *k* is the measurement sampling parameter. Based on the collect data and the regression coefficient ${\{\left(y\left(p_{k}\right),\phi \;\left(p_{k}\right)\right)\}}_{k=1}^{n}$, the objective is to search for the $\hat{\Theta }$ that minimizes the least-squares error:

(19) $$\sum_{k=1}^{n}{\left|y\left(p_{k}\right)-\phi^{T}\left(p_{k}\right)\hat{\Theta }\right|}^{2}$$

The goal of this learning process is to minimize the difference between the observed data and the predictions, effectively seeking the parameter estimate $\hat{\Theta }$ that reduces the least-squares error.

#### 3.1.1 Noiseless measurements

We consider the model in Eq. (2) without the noise w(k). The spatial estimation algorithm based on noise level can be used to the minimum variance of the estimation error [41]. For a set ${\{\left(y\left(p_{k}\right),\phi \;\left(p_{k}\right)\right)\}}_{k=1}^{n}$, the error function in Eq. (14) is minimized by the optimal least squares estimation solution as follow:

(20) $$\hat{\Theta }\left(n\right)=P\left(n,1\right)\Phi^{T}\left(n,1\right)Y\left(n,1\right)$$

As described in Eq. (1), in the process of collective learning, as time progresses from *t* to t+△t, each micro/nanorobot collects a large amount of measurement sample data from micro/nanorobot discovery and the shared information of their group [42]. If we consider the data previously collected up to the last iteration as ${\{\left(y\left(p_{k}\right),\phi \;\left(p_{k}\right)\right)\}}_{k=1}^{n-s}$, where *n* - s is a collection of past data, then the current understanding of the potential field $\hat{F}\left(\cdot \right)$ is updated by adding the new collective measurements. Suppose $\Phi^{T}\left(t\right)\Phi \left(t\right)$ is nonsingular for all *t*. With this foundation, the recursion algorithm for the newly collected observational data and the associated regressors ${\{\left(y\left(p_{k}\right),\phi \;\left(p_{k}\right)\right)\}}_{k=n-s+1}^{n}$ as follow:

(21) $$\begin{array}{c} K\left(n\right)=P\left(n-s\right)\Phi_{*}^{T}{\left[I_{s}+\Phi_{*}P\left(n-s\right)\Phi_{*}^{T}\right]}^{-1}\\ P\left(n\right)=\left[I_{m}-K\left(n\right)\Phi_{*}\right]P\left(n-s\right)\\ \hat{\Theta }\left(n\right)=\hat{\Theta }\left(n-s\right)+K\left(n\right)\left[Y_{*}-\Phi_{*}\hat{\Theta }\left(n-s\right)\right]\\ \hat{F}\left(p\right)=\phi^{T}\left(p\right)\hat{\Theta }\left(n\right) \end{array}$$

To simplify notation, let $Y_{*}=Y\left(n,n-s+1\right)\in R^{s}$, $\Phi_{*}=\Phi \left(n,n-s+1\right)\in R^{s\times m}$, $\Phi^{T}=\Phi^{T}\left(n,1\right)\in R^{n\times m}$, $Y\left(n\right)=Y\left(n,1\right)\in R^{n}$ and $P\left(n\right)=P\left(n,1\right)\in R^{m\times m}$. The recursive estimation in Eq. (21) can be expressed as the least square estimation that minimizes the error function in Eq. (22).

#### 3.1.2 Noisy measurements

For *n* < *m*, $\Phi^{T}\left(n\right)\Phi \left(n\right)$ is always singular. *W* is nonsingular for n≥m, unless measured at only one set of measurement zeros, e.g., bisecting two gaussian radial basis functions such that $\phi_{i}\left(p\right)=\phi_{j}\left(p\right)$. In the initial state of $\hat{\Theta }\left(0\right)$ and P ≻ (0), each micro/nanorobot uses the initial parameter distribution for a recursive LSE algorithm in Eq. (21). This process reflects the micro/nanorobots relying on prior information when interacting with the environment [43]. As new observations emerge, micro/nanorobots continuously use recursive algorithms to update and enhance their perception of the potential field. We have:

(22) $$P^{-1}\left(n\right)=P^{-1}\left(0\right)+\Phi^{T}\left(n\right)\Phi \left(n\right)≻0$$

Next, consider the interaction model (2), where each micro/nanorobot is affected by noise, denoted by *w*(*k*). The noise w(k) is defined as a sequence of white noise with unknown variance *W*.

(23) $$E\left(w\left(k\right)\right)=0,E\left(w\left(k\right)w\left(l\right)\right)=\left\{ \begin{array}{c} W>0\;if\;k=l\\ 0\;if\;k\neq l \end{array}\right.$$

where E is defined as the expectation operator. We suppose the existence of L<∞ such that

(24) theprobabilityof|w(k)|<Lisone(w.p.1)∀k

Let the estimation error vector denoted by $\hat{\Theta }\left(n\right)=\hat{\Theta }\left(n\right)-\Theta$. At the location p∈M, the error of the estimated potential field is defined as:

(25) $$\hat{F}\left(S,p\right)=\hat{F}\left(S,p\right)-F\left(p\right)=\phi^{T}\left(p\right)\tilde{\Theta }\left(\left|S\right|\right)$$

where |S| is the cardinality of the set S. At p∈M, the error of the estimated potential field is given:

(26) $$\hat{F}\left(S,p\right)=E(\hat{F}\left(S,p\right)+\in \left(S,p\right)$$

where

$$E\left(\hat{F}\left(S,p\right)\right)=\phi^{T}\left(p\right)\left[P\left(\left|S\right|\right)\sum_{p_{t}\in S}\phi \left(p_{t}\right)\phi^{T}\left(p_{t}\right)-I_{m}\right]\Theta$$

$$\epsilon \left(S,p\right)=\phi^{T}\left(p\right)\left[P\left(\left|S\right|\right)\sum_{t=1}^{\left|S\right|}\phi \left(p_{t}\right)w\left(t\right)\right]$$

where |S| represents the aggregate sum of all measurement data. For the continuous motivation coordination strategy, identified by the condition $\left(\Phi_{*}^{T}\Phi_{*}≻0\right)$, the estimator remains asymptotically unbiased.

(27) $$\lim_{\left|S\right|\to \infty }E\left(\hat{F}\left(S,p\right)\right)=0,\forall v\in M$$

The covariance matrix $E(\in \left(S,p\right)\in {\left(S,p\right)}^{T}$ is defined as:

(28) $$\phi^{\prime T}\left(p\right)\frac{W}{\left|S\right|}R^{-1}\left(S\right)\phi^{\prime }\left(p\right)$$

where *R*(*S*) is represented as:

(29) $$R\left(S\right)=\left[\frac{P^{-1}\left(0\right)}{\left|S\right|}+\frac{1}{\left|S\right|}\sum_{v_{k}\in S}\phi \left(p_{k}\right)\phi^{T}\left(p_{k}\right)\right]$$

R(S) acts asymptotically as the time average of the outer product of the set of basis functions, calculated at the measurement point *S*. Next, the cooperative control protocol is proposed.

### 3.2 Cooperative control

In the context of micro/nanorobot swarm applications, these robots need to navigate complex and variable terrains, such as human tissues or blood vessels. A recursive algorithm is required for potential field estimation and cooperative control. The micro/nanorobots in the swarm update their estimated potential field gradient using this recursive algorithm and determine their cooperative control strategy based on neighbor measurements [44]. This approach allows each micro/nanorobot to iteratively improve its understanding of the local potential field, which is essential for navigation in unpredictable and potentially dangerous environments. This algorithm introduces a new time symbol for coordination, replacing t∈Z with $n-s\in Z_{\geq 0}$ and $t+1\in Z_{\geq 0}$ with $n\in Z_{\geq 0}$ in (16). At location $q_{i}\left(t\right)$, the recursive algorithm has a new time index for micro/nanorobot *i* is defined as:

(30) $$\begin{array}{c} K_{i}\left(t+1\right)=P_{i}\left(t\right)\Phi_{*i}^{T}{\left(I_{s}+\Phi_{*i}P_{i}\left(t\right)\Phi_{*i}^{T}\right)}^{-1}\\ P_{i}\left(t+1\right)=\left(I_{m}-K_{i}\left(t+1\right)\Phi_{*i}\right)P_{i}\left(t\right)\\ {\hat{\Theta }}_{i}\left(t+1\right)={\hat{\Theta }}_{i}\left(t\right)+K_{i}\left(t+1\right)\left[Y_{*i}-\Phi_{*i}{\hat{\Theta }}_{i}\left(t\right)\right]\\ \nabla {\hat{F}}_{i}\left(t,q_{i}\left(t\right)\right)=\phi^{\prime T}\left(q_{i}\left(t\right)\right){\hat{\Theta }}_{i}\left(t+1\right) \end{array}$$

Where $\nabla {\hat{F}}_{i}\left(t,p\right):Z_{\geq 0}\times M⟶R^{2}$ denotes the gradient of the estimated potential field at position *ν*, as determined by measurements collected before the time *t* + 1. $Y_{*i}$ and $\phi_{*i}$ for an micro/nanorobot *i* are defined similarly to $Y_{*}$ and $\phi_{*}$ in Eq. (16). $Y_{*i}$ represents the aggregated data obtained through cooperative measurement within the group. For all j∈N(i,q(t))∪{i}, we obtained:

(31) $$Y_{*i}=\Phi_{*i}\Theta +\left[\begin{array}{c} \vdots \\ w_{j}\left(k\right)\\ \vdots \end{array}\right]=\Phi_{*i}\Theta +w_{*i}\left(t\right)$$

where, during the period from time *t* to *t* + 1, micro/nanorobots collect data using *t* as a reference point. $w_{j}\left(k\right)$ means that each micro/nanorobot receives noise. Next, we introduce a new variable *w*_∗*i*_ (*t*) for subsequent analyses. Based on this, a distributed control *p*_*i*_ (*t* + 1) is designed for each micro/nanorobot *i*:

(32) $$p_{i}\left(t+1\right)=\frac{\gamma \left(t+1\right)}{\Delta_{t}}\left[\frac{\Delta_{t}}{\gamma \left(t\right)}p_{i}\left(t\right)+\gamma \left(t\right)u_{i}\left(t\right)\right]$$

with

(33) $$u_{i}\left(t\right)=-\nabla U\left(q_{i}\left(t\right)\right)+k_{4}\nabla {\hat{F}}_{i}\left(t,q_{i}\left(t\right)\right)-k_{di}\frac{\Delta_{t}}{\gamma \left(t\right)}p_{i}\left(t\right)+\sum_{j\in N\left(i,q\left(t\right)\right)}a_{ij}\left(q\left(t\right)\right)\left(\frac{\Delta_{t}\left(p_{j}\left(t\right)-p_{i}\left(t\right)\right)}{\gamma \left(t\right)}\right)$$

where $k_{4}\in R_{>0}$ is the gain factor for the estimated gradient of the potential field. $k_{di}\in R_{\geq 0}$ is a gain of the speed feedback, which is convenient for dynamic motion adjustment. $-\nabla U\left(q_{i}\left(t\right)\right)$ is the gradient of the artificial potential described in Eq. (15), and its role is to generate attractive and repulsive forces between micro/nanorobots. The second item in Eq. (33) represents the damping force. Because it regulates the momentum of the micro/nanorobot to ensure a smooth control of the process, rather than sudden movement. The third term is the velocity of an micro/nanorobot, aligning it with the velocities of their neighbors. The last term is the gradient ascent of the estimated field, which is used to guide micro/nanorobots toward the peak of the potential field based on their evolving estimates. Furthermore, to better search for the maximum peak of the potential fields, it is necessary to gradually reduce the control intensity of the micro/nanorobot. This is achieved through a control protocol with a standard adaptive gain sequence γ(t), as introduced in Eq. (32). Adaptive gain sequences enable micro/nanorobots to remain responsive to the changing potential fields, thus providing dynamic methods for collective navigation and decision-making [45]. The control protocol needs to satisfies the following conditions:

(34) $$\begin{array}{c} \gamma \left(t\right)>0,\sum_{t=1}^{\infty }\gamma \left(t\right)=\infty ,\sum_{t=1}^{\infty }\gamma^{2}\left(t\right)=<\infty \\ \lim_{t\to \infty }\;\sup\left[1/\gamma \left(t\right)-1/\gamma \left(t-1\right)\right]<\infty \end{array}$$

The gain sequences are often utilized in random approximation algorithms and for analyzing convergence through the ordinary differential equation method [46,47]. To conduct this analysis effectively [48], a transformation of the variables *z*_*i*_ (*t*) is introduced, which represents a new form of the velocity state *v*_*i*_ (*t*), we obtain:

(35) $$z_{i}\left(t\right)=\frac{\Delta_{t}}{\gamma \left(t\right)}v_{i}\left(t\right)$$

where *v*_*i*_(*t*) represents the control input of the micro/nanorobot *i.* As the variable in (35) changes, the dynamics of micro/nanorobot *i* is expressed as follows:

(36) $$\left\{ \begin{array}{c} q_{i}\left(t+1\right)=q_{i}\left(t\right)+\gamma \left(t\right)z_{i}\left(t\right)\text{,}\\ z_{i}\left(t+1\right)=z_{i}\left(t\right)+\gamma \left(t\right)u_{i}\left(t\right)\text{,} \end{array}\right.$$

where $\Delta_{t}v_{i}\left(t\right)$ is replaced by $\gamma \left(t\right)p_{i}\left(t\right)$, $t+\Delta_{t}\in R_{\geq 0}$ is replaced by $t+1\in Z_{\geq 0}$, $t\in R_{\geq 0}$ is replaced by $t\in Z_{\geq 0}$, and the new symbol is applied to Eq. (1). Combined with the control proposed in Eqs. (32), (33) and the discrete time model in Eq. (36) as follows:

(37) $$\begin{array}{c} q_{i}\left(t+1\right)=q_{i}\left(t\right)+\gamma \left(t\right)z_{i}\left(t\right)\\ z_{i}\left(t+1\right)=z_{i}\left(t\right)+\gamma \left(t\right)\{-\nabla U\left(q_{i}\left(t\right)\right)-k_{di}z_{i}\left(t\right)\\ \;-\nabla \Psi_{G}\left(z_{i}\left(t\right)\right)+k_{4}\phi^{T}\left(q_{i}\left(t\right)\right){\hat{\Theta }}_{i}\left(t+1\right)\} \end{array}$$

where ∇*Ψ*_*G*_(*p*_*i*_(*t*)) is the gradient of the inconsistent function relative to *z*_*i*_:

$$\nabla \Psi_{G}\left(z_{i}\left(t\right)\right)=\sum_{j\in N\left(i,q\left(t\right)\right)}a_{ij}\left(q\left(t\right)\right)\left(z_{i}\left(t\right)-z_{j}\left(t\right)\right)$$

The complex system is transformed into a recursive stochastic algorithm with states in section 4. In conclusion, the sensing capabilities of each micro/nanorobot are crucial for the swarm's adaptability and responsiveness to dynamic environments. These capabilities allow each robot to gather critical information from its surroundings. This information is then shared within the swarm, enhancing collective decision-making and navigation. This process ensures that the swarm can dynamically adjust to environmental changes and navigate effectively toward therapeutic targets.

## 4 Convergence analysis

In order to study the convergence of Eq.s (41), (48), (45) the ordinary differential equation method is used [47,49]. This method not only analyzes the convergence tendency based on micro/nanorobot equations, but also provides support for collective behavior.

(38) x(t)=x(t−1)+γ(t)Q(t;x(t−1),ϕ(t))

and the observation process

(39) ϕ(t)=g(t;x(t−1),ϕ(t−1),e(t))

In order to implement the ordinary differential equation method for nonlinear observation processes described in (39), it is essential to satisfy specific regularity conditions [46]. In the implementation of algorithms, it is common practice to introduce projection or saturation to satisfy the necessary bounded conditions in the ordinary differential equation method [49]. The use of projection or saturation not only satisfies the bounded premise of the ordinary differential equation method, but also ensures that micro/nanorobot behavior remains within predetermined constraints. Since the movement of the micro/nanorobot is guided by a single integrator model, its positioning can be controlled as follows:

(40) $$q_{i}\left(t+1\right)=q_{i}\left(t\right)+\gamma \left(t\right)p_{i}\left(t\right)$$

where *p*_*i*_ (*t*) denotes the control. Then the usual saturation given by [·]_D_ can be applied:

(41) $$x\left(t\right)={\left[\Omega \left(t\right)\right]}_{D}=\left\{ \begin{array}{c} \Omega \left(t\right),\Omega \left(t\right)\in D\\ x\left(t-1\right),\Omega \left(t\right)\notin D \end{array}\right.$$

where D is the compact principal of *D*_*R*_ and its regularity condition holds. x(t) and Ω(t) represent the left and right sides of Eq. (38). Eq. (38) can be transformed into a standard form based on the closed-loop system presented in Eq. (37). Then, to verify the regularity conditions, the assumptions is given as:

M1 A number s ≥ m of measurement values can be collected by the agent and its neighbors at the location ${\left\{p_{k}\right\}}_{k=1}^{s}$, $\sum_{k=1}^{n}\phi \left(p_{k}\right)\phi^{T}\left(p_{k}\right)≻0$, where *k* is in Eq. (16).

M2 The adjacency matrix and the artificial potential force are continuously differentiable for q, and the derivatives are bounded.

M3 The projection algorithm (42) can be used to coordinate the algorithm (38). Let *D* in (41) be defined as a convex compact set by $D=M^{Ns}\times M_{p},where\;M_{p}={\left[p_{\min},p_{\max}\right]}^{{2N}_{s}}$.

The prerequisites for effective adaptive control are discussed, emphasizing the need for continuous excitation (M1), behavioral adjustments (M2), and boundedness (M3) in the context of searching for potential fields and controlling micro/nanorobots movements.

Lemma 1 The algorithms (38), (39), and (41) are to be considered, and they are subject to the regularity conditions [47]. An open connected subset of D_S_ is denoted as D_R_. In Eq. (41), D is defined as the compact subset of D_R_, and it is characterized by the fact that the trajectories of the associated ordinary differential equation are subject to this definition.

(42) $$\frac{d}{d\tau }x\left(\tau \right)=f\left(x\left(\tau \right)\right)$$

where $f\left(x\right)=\lim_{t\to \infty }\to EQ\left(t;x,\bar{\phi }\left(t,x\right)\right)\text{,}$ for τ > 0, the starting point in *D* can always stay in the closed subset ${\bar{D}}_{R}$ of *D*_*R.*_ Suppose that the differential Eq. (42) has a domain of attraction *D*_*A*_ ⊃ *D* and is an invariant set *D*_*c*_. Then either

(43) $$x\left(t\right)\;\to \;D_{c},probability\;one\;is\;t\;\to \infty \text{,}$$

or

(44) x(t)→∂D,probabilityoneist→∞,

where ∂*D* is the boundary of *D*. The conclusion (44) can only be established when the trajectory of the differential equation in (42) leaves *D* in Eq. (44).

Lemma 2 Under the assumption of condition M1-M3, the transformation recursive algorithm follows the constraint of regularity condition and $\left(M^{N_{S}}\backslash Z\right)\times M_{P}\subset D\subset D_{R}$, where $M_{P}={\left[p_{\min},p_{\max}\right]}^{{2N}_{s}}$ and Z are represented by:

(45) $$Z=\left\{q\in M^{N_{s}}|\sum_{j\in \left\{i\right\}\cup N\left(i,q\right)}\phi \left(q_{j}\right)\phi^{T}\left(q_{j}\right)⊁0,\forall i\in l\right\}$$

Moreover, *f* (*x*) in (40) of Lemma 1 is defined as follows:

(46) $$f\left(x\right)=\left[\begin{array}{c} p\\ -\nabla U\left(q\right)-\left(\hat{L}\left(q\right)+K_{d}\right)p-\nabla C\left(q\right) \end{array}\right]$$

where C(q)∈R≥0 denotes the collective performance cost function as follows:

(47) $$C\left(q\right)=k_{4}\sum_{i\in l}\left[c_{\max}‐c\left(q_{i}\right)\right]$$

where $c_{\max\;}$ is the maximum of the of potential field and is assumed to be bounded.

Proof Regularity conditions is validated as follows:

C1 The measurement noise hypothesis in (28) and (29) and lemma 2 in M1 and M3 are satisfied.

C2 This is satisfied by assuming that the radial basis functions in M2 and $\nabla \hat{C}\left(\phi ,q\right)$ are smooth and bounded derivatives relative to q.

C3 A and B are smooth in $D_{R}$ because of they are functions of smooth radial basis functions.

C4 We introduce the argument [50].

$$\phi \left(t\right)-\bar{\phi }\left(t\right)={A\left(t;x\right)|}_{\tilde{x}\left(t-1\right)}\left(\phi \left(t-1\right)-\bar{\phi }\left(t-1\right)\right){+}_{{\frac{\partial g}{\partial x}|}_{\begin{array}{c} \bar{x}\left(t-1\right)\\ \bar{\phi }\left(t-1\right) \end{array}}}\left(x\left(t-1\right)-\bar{x}\right){=}_{{\frac{\partial g}{\partial x}|}_{\begin{array}{c} \tilde{x}\left(t-1\right)\\ \tilde{\phi }\left(t-1\right) \end{array}}}\left(x\left(t-1\right)-\bar{x}\right){+}_{{\sum_{i=2}^{t-n}\frac{\partial g}{\partial x}|}_{\begin{array}{c} \tilde{x}\left(t-1\right)\\ \tilde{\phi }\left(t-1\right) \end{array}}}\left(x\left(t-i\right)-\bar{x}\right)\left({\prod_{j=1}^{i-1}A\left(t;x\right)|}_{\tilde{x}\left(t-1\right)}\right)$$

where the mean value theorem is applied to the smoothing *g* of the related x and ϕ. ${\left[{\tilde{x}}^{T}\left(s\right),{\tilde{\phi }}^{T}\left(s\right)\right]}^{T}$ is the point between ${\left[x^{T}\left(s\right),\phi^{T}\left(s\right)\right]}^{T}$ and ${\left[{\tilde{x}}^{T}\left(s\right),{\tilde{\phi }}^{T}\left(s\right)\right]}^{T}$.

Consequently,

$$\left\|\phi \left(t\right)-\bar{\phi }\left(t\right)\right\|\leq {\|}_{{\frac{\partial g}{\partial x}|}_{\begin{array}{c} \tilde{x}\left(t-1\right)\\ \tilde{\phi }\left(t-1\right) \end{array}}}\|\left\|x\left(t-1\right)-\bar{x}\right\|+\sum_{i=2}^{t-n}\left(\prod_{j=1}^{i-1}\left\|{A\left(t;x\right)|}_{\tilde{x}\left(t-j\right)}\right\|\left\|{\frac{\partial g}{\partial x}|}_{{\tilde{x}\left(t-i\right)}_{\begin{array}{c} \tilde{\phi }\left(t-i\right) \end{array}}}\right\|\left\|x\left(t-i\right)-\bar{x}\right\|\right)\leq \sum_{i=1}^{t-n}{\left(1-\delta \right)}^{i-1}\max_{n\leq s\leq t}\left\|\frac{\partial g}{\partial x}\left(s\right)\right\|\max_{n\leq s\leq t}\left\|x\left(s\right)-\bar{x}\right\|<\frac{1}{\delta }C\max_{n\leq s\leq t}\left\|x\left(s\right)-\bar{x}\right\|<C\max_{n\leq s\leq t}\left\|x\left(n\right)-\bar{x}\right\|$$

C5 For a fixed $\bar{x}$, we have:

$${\bar{\phi }}_{i}\left(t,\bar{x}\right)=\prod_{k=s+1}^{t}A\left(k;\bar{x}\right){\bar{\phi }}_{i}\left(s,\bar{x}\right)+\sum_{j=s+1}^{t}\left[\prod_{k=j+1}^{t}A\left(k;\bar{x}\right)\right]B\left(j;\bar{x}\right)e\left(j\right),i\in \left\{1,2\right\}\text{.}$$

Under M1 and M3, $A\left(k;\;\bar{x}\right)k<\lambda \left(\bar{x}\right)$ for all k∈{s+1,...,t}, where $\lambda \left(\bar{x}\right)<1$. Therefore, the following can be obtained:

$\left\|{\bar{\phi }}_{1}\left(t,\bar{x}\right)-{\bar{\phi }}_{2}\left(t,\bar{x}\right)\right\|<\lambda^{t-s}\left(\bar{x}\right)\left\|{\bar{\phi }}_{1}\left(s,\bar{x}\right)-{\bar{\phi }}_{2}\left(s,\bar{x}\right)\right\|\text{,}$ for all $\bar{x}\in \left(M^{Ns}\setminus Z\right)\times M_{p}\subset D_{s}$, where *Z* is defined by (45) as a set.

C6 In Eq. (44), the elements of *Q* are defined as deterministic functions of $x\in D_{R}$, with the exception of $\nabla \hat{C}\left(\phi \left(t\right),q\right)$. This is due to the application of the principles in M1 and Eq. (38). When we consider a specific value of *q*, it can be obtained: $\lim_{t\to \infty }E\left(\nabla \hat{C}\left(\phi \left(t\right),q\right)\right)=\nabla C\left(q\right)$, which C6 and (27) were proved.

C7 Satisfaction of this condition is based on the assumption of measurement noise, which is detailed in Eq. (28).

The conditions C8, C9, C10, and C11 are fulfilled through the application of the time-varying gain sequence, as shown in Eq. (23). Finally, the micro/nanorobot needs to minimize the global performance cost as follows:

(48) $$V\left(q\left(\tau \right),p\left(\tau \right)\right)=C\left(q\left(\tau \right)\right)+F\left(q\left(\tau \right)\right)+\frac{p^{T}\left(\tau \right)p\left(\tau \right)}{2}$$

where *C*(*q*) represents the cost function associated with the position of the micro/nanorobot *q*. It is designed to incentivize the micro/nanorobot to track the peak of the potential field. *F*(*q*) is a cost function related to *q*, which contains strategies for obstacle avoidance and collective behavior. The last term on the right side of (48) represents the kinetic energy of the complex system and is a separate cost function of *p*.

## 5 Simulation results

In this study, we have developed a conceptual framework aimed at simulating micro/nanorobots for targeted therapy and drug delivery in dynamic biomedical environments based on potential fields mechanisms. The potential field mechanism enables micro/nanorobots to navigate to treatment targets while avoiding collisions and optimizing their paths. This optimization approach ensures efficient and precise drug delivery and targeted therapy. Local interactions are a key aspect of this framework. The micro/nanorobots share information and adjust their positions based on local sensing, similar to the gradient descent steps in optimization algorithms. This local interaction ensures that the micro/nanorobots respond promptly to changes and disturbances, maintaining smooth and coordinated movement towards the targets. In addition, the global objective in the simulation is to minimize the mean squared error (MSE), ensuring that the swarm collectively achieves optimal positioning and task execution. By continuously minimizing the MSE, the micro/nanorobots improve their accuracy in reaching therapeutic targets. This process is analogous to an optimization algorithm where the goal is to find the minimum of an objective function. By incorporating distributed learning, the simulation ensures that the swarm of micro/nanorobots can collectively respond to disturbances and maintain coordination. This distributed approach enhances their ability to optimize their movements and strategies in a decentralized manner, reflecting the principles of swarm intelligence often used in optimization algorithms. As a result, the micro/nanorobots exhibit improved coordination and precision in reaching their therapeutic targets.

The initial conditions for the micro/nanorobots include fixed starting positions and random distribution within the monitoring area, designed to reflect the natural variability and complexity present in biological systems. We analyze the simulation results to understand the behavior of these micro/nanorobots in dynamic biomedical environments and to explore the effects of different parameters and conditions.

### 5.1 Standard environment

**Fig. 2:**
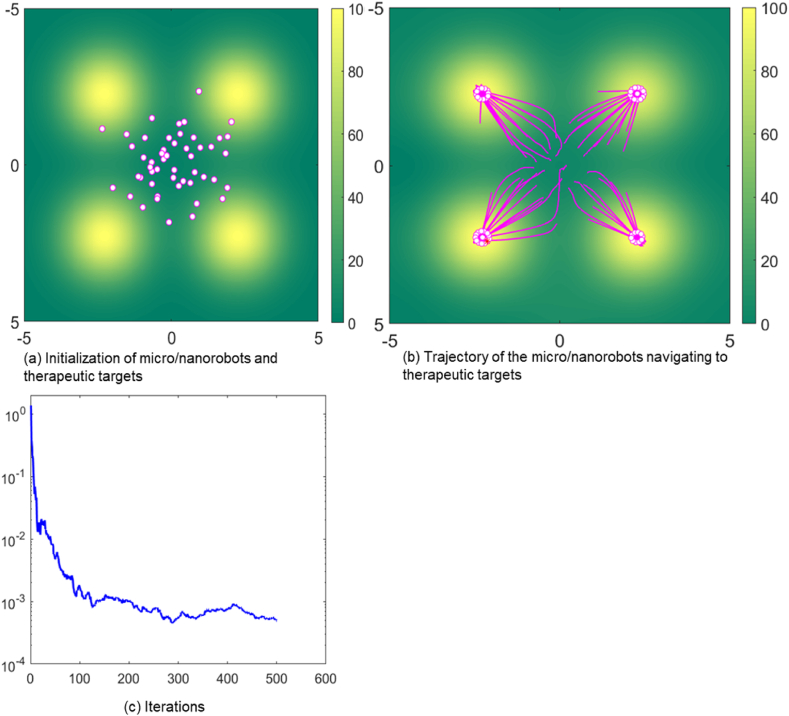
Trajectory of micro/nanorobots navigating to therapeutic targets in a standard environment.

In the initial simulation detailed shown in Fig. 2(a), the white dots represent the current position of a single micro/nanorobot, setting up a group of 50 micro/nanorobots. These micro/nanorobots are intelligently designed to traverse complex internal environments autonomously. Yellow areas indicate potential fields, meaning areas with higher concentrations of pathological cells or areas requiring concentrated drug delivery. The intensity of yellow indicates that these locations have the largest potential field peaks and have a greater impact on the navigation control of micro/nanorobots, attracting them with higher forces. Four potential fields are set up in the simulated environment, which represent targets for targeted therapy or drug delivery. The micro/nanorobots are guided by potential fields to move towards these therapeutic targets. The pink tracks depict the path of the micro/nanorobots toward the therapeutic target. A background gradient of green to yellow tones indicates a chemical attractant or heat distribution in the body. The noise level parameter *W* is set to 1 to reflect the complexity and uncertainty that these micro/nanorobots may encounter in a biomedical environment, such as fluctuating biological conditions, physiological barriers, and dynamic tissue properties. The equilibrium distance D is set to 0.6, which represents the ideal distance maintained between these micro/nanorobots, optimizing their collective efficacy in navigating to therapeutic targets in the biomedical environment.

As shown in Fig. 2 (b), all micro/nanorobots navigate to four therapeutic targets. The micro/nanorobots use distributed learning to overcome environmental disturbances and achieve collective precision when reaching a therapeutic targets. The convergence of micro/nanorobots under noisy conditions indicates the stability of micro/nanorobots in collective behavior. The principle of attraction and repulsion between the micro/nanorobots is crucial for maintaining their coordinated movement and ensuring they can effectively reach their therapeutic targets. The artificial potential energy function plays a significant role in this coordination by creating forces that either attract or repel the robots based on their positions relative to each other and the potential fields. The pink tracks in the simulation illustrate how the micro/nanorobots dynamically adjust their paths in response to the potential fields and the positions of other robots. As they move towards the therapeutic targets, their paths are influenced by the continuous interplay of attractive and repulsive forces, ensuring that they converge on the targets efficiently while avoiding collisions. By maintaining the equilibrium distance and responding dynamically to the potential fields and environmental gradients, the micro/nanorobots exhibit a high degree of coordination and adaptability. This enables them to navigate complex environments and effectively reach their therapeutic targets, demonstrating the potential of these intelligent systems in biomedical applications.

In Fig. 2(c), the blue line represents the mean square error (MSE). The MSE is a crucial indicator for evaluating the performance and precision of the micro/nanorobots in navigating towards their therapeutic targets. It quantifies the average squared deviation of each micro/nanorobot's actual position from its intended target position. By calculating the squared difference between the actual and target positions, MSE captures the magnitude of the error for each micro/nanorobot. Thus, averaging these squared errors across all micro/nanorobots yields a single comprehensive value, representing the overall precision and effectiveness of the swarm's navigation. A lower MSE indicates higher precision, showing that the micro/nanorobots are closer to their target positions and demonstrating effective navigation and coordination. It also reflects the micro/nanorobots' ability to adapt successfully to environmental disturbances, maintaining their course towards the therapeutic targets. Improved coordination among the swarm is evident when the MSE is low, indicating that the robots are working together efficiently to reach their goals. Conversely, a higher MSE suggests that the micro/nanorobots are experiencing difficulties in maintaining their paths, possibly due to environmental challenges or inefficiencies in their coordination mechanisms. Therefore, monitoring and minimizing the MSE is essential for ensuring the successful deployment of micro/nanorobots in complex biomedical environments. This focus on reducing MSE enhances the collective proficiency of the swarm, ensuring precise navigation and effective therapeutic interventions.

Furthermore, the lack of significant oscillatory motion near the target peak indicates that the group has reached critical equilibrium. Fig. 2(c) shows the trajectory of the MSE during the movement of the swarm micro/nanorobot, highlighted by a significant decline in MSE as depicted by the blue line. This rapid decrease in MSE signals a phase of rapid adaptation or learning. This shows that micro/nanorobots are rapidly improving their coordination methods to precisely assess the biomedical environment and efficiently navigate to specific therapeutic targets. As the simulation progresses towards the 500th iteration, the reduction in MSE starts to plateau, indicating that the swarm of micro/nanorobots is fine-tuning their collective movements and strategies to select the most efficient pathway and delivery mechanism. The achievement of a minimal MSE value around the 500th iteration reflects the micro/nanorobots' collective proficiency in rectifying earlier navigational inaccuracies. This accomplishment enhanced their group's ability to synchronize their actions towards the successful achieve the specified delivery point.

### 5.2 Noise environment

**Fig. 3:**
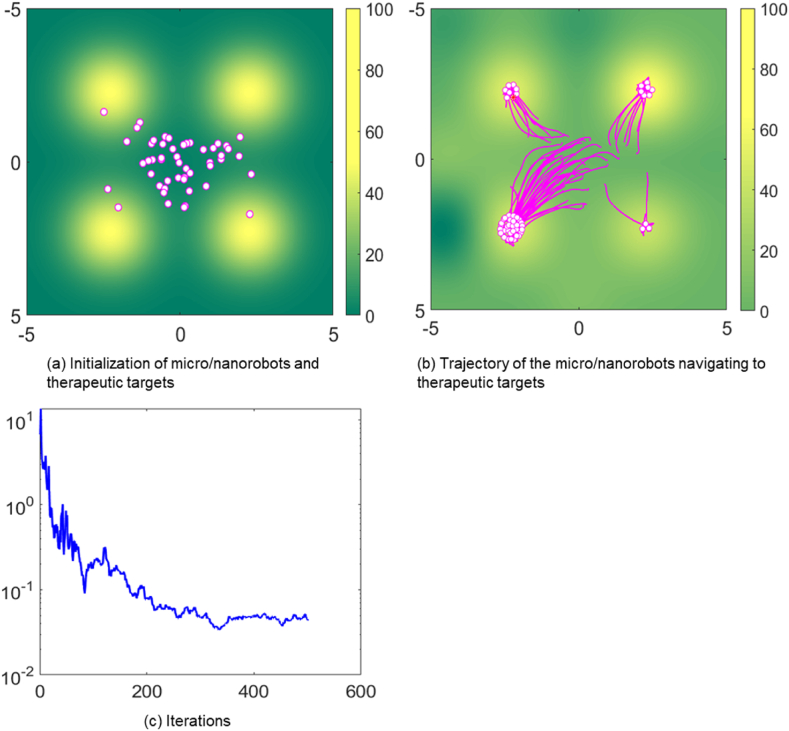
Trajectory of micro/nanorobots navigating to therapeutic targets in noisy environment.

Fig. 3 depict the simulation of 50 micro/nanorobots in an environment heavily affected by disruptive factors, marked by the noise level (*W* = 10). A noise level of 10 means that the micro/nanorobot is severely disturbed by the signal. This may include a range of physiological noise sources, such as fluid turbulence caused by blood flow, heterogeneity in tissue density, and bio electrochemical noise from cellular and extracellular activities. These factors can potentially interfere with the sensory and propulsion systems of micro/nanorobots, thereby posing significant challenges to navigation. As shown in Fig. 3(b), When the micro/nanorobots approach the therapeutic target, their distribution is significantly uneven. Specifically, there is a higher accumulation of robots in the lower-left target area, suggesting a denser engagement of the therapeutic delivery in that area. In contrast, the lower-right region showed a low number of micro/nanorobots and a low amount of therapeutic drug delivery to that site.

Fig. 3(c) shows the fluctuations of the MSE over time, indicating that stability is decreasing in a noise-filled environment. This instability means that it is difficult for micro/nanorobots to maintain effective communication and precise navigation under noise interference. Thus, the graph illustrates the negative correlation between elevated initial noise parameters and the ability to explore therapeutic targets. Despite the substantial noise, the directional path of the nanorobots toward the target indicates that their distributed learning and control algorithms are highly resilient and adaptive. This suggests that micro/nanorobots have the ability to compensate for environmental variables and perturbations through local interactions, maintaining their course and functional objectives with a high degree of accuracy.

### 5.3 Density environment

**Fig. 4:**
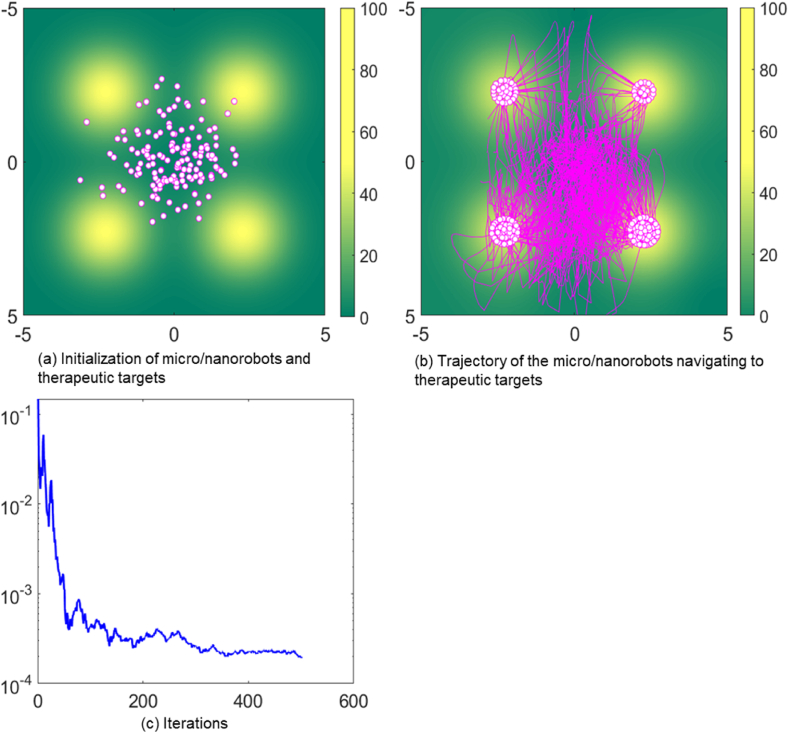
Trajectory of micro/nanorobots navigating to therapeutic targets in a density environment.

Fig. 4 shows the action efficiency of swarm micro/nanorobots at high density. In Fig. 4 (a), with the group size is 150 micro/nanorobots and the noise parameter is set to 1, the efficiency of collective search for therapeutic targets decreases as the number of micro/nanorobots increases. In Fig. 4 (b), as the density increases, the micro/nanorobots interfere with each other, which hinders the rapid and accurate targeting and release of therapeutic drugs. Moreover, the high density of micro/nanorobots will also increase the complexity of information exchange, which will reduce the coordination and response speed of the system. Because they require the rapid and accurate targeting and release of drugs to maximize treatment impact and minimize adverse effects.

Fig. 4 (c) shows the effect of population density on the operational efficiency of micro/nanorobots. We can see an inverse relationship between population density and therapeutic effectiveness, where an increase in the number of micro/nanorobots leads to interference between individuals, reducing the precision and efficiency of drug delivery to the disease site. In addition, the trend in MSE indicates that the rate at which consensus is reached between groups is decreasing, thus potentially slowing down the collective decision-making process. Therefore, in the practice of targeted therapy and drug delivery, there is a need to balance the number and density of micro/nanorobots to maximize therapeutic effectiveness and reduce potential side effects.

### 5.4 In a shortened communication environment

**Fig. 5:**
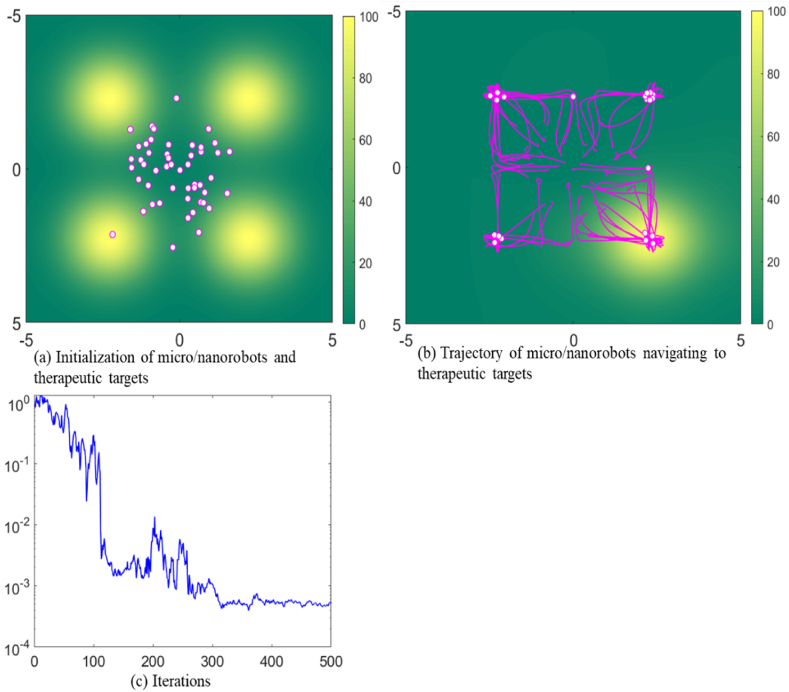
Trajectory of micro/nanorobots navigating to therapeutic targets a shortened communication environment.

Fig. 5 (b) demonstrates the critical role of local interaction and the communication radius among micro/nanorobots in their navigation towards therapeutic targets. In the simulation, when the communication radius is reduced to 0.4, which is 10 times shorter than the initial setting, significant changes in the robots' behavior and functionality are observed. The primary effect of shortening the communication radius is the weakening of local interaction and information-sharing capabilities. Micro/nanorobots rely on their ability to communicate and coordinate their movements to precisely navigate towards potential field peaks. A reduced communication radius impairs this precision, ultimately affecting the accuracy and effectiveness of targeted therapies and drug delivery. Another critical consequence of a shortened communication radius is the loss of group cohesion among the micro/nanorobots. With a diminished interaction radius, the micro/nanorobots lose this cohesion, resulting in a disjointed and less effective swarm. This lack of coordination significantly reduces the efficiency of their collective behavior, hindering their ability to function as an integrated unit. In summary, maintaining an adequate communication radius is essential for ensuring effective local interaction, precise navigation, and successful targeted therapies. The lack of an effective interaction radius leads to significant drawbacks, including reduced treatment success, loss of group cohesion, and an inability to leverage the collective intelligence of the micro/nanorobot swarm.

As shown in Fig. 5(c), the initial convergence curve starts at a higher error value. With a limited range for local interaction, the micro/nanorobots struggle to share information effectively and coordinate their movements from the outset. This lack of effective communication results in significant variability and error in their initial positions and states. As time progresses, a steep decline in the error value marks the rapid initial convergence phase. During this phase, the micro/nanorobots quickly adjust their movements to better coordinate with one another despite the communication challenges. Around the 100th iteration, the curve transitions to a more gradual decline, indicating a second phase of convergence where the adjustments become finer. The most notable aspect of the convergence curve occurs between iterations 200 and 300, where fluctuations are also observed. This reflecting the challenges the robots face in adapting to dynamic environmental conditions and interacting with each other. Beyond the 300th iteration, the convergence curve stabilizes and reaches a lower level. This stabilization indicates that, despite the reduced communication radius, the micro/nanorobots eventually achieve a stable state with minimal error. Ensuring an adequate communication radius is essential for minimizing variability, achieving smoother convergence, and enhancing the overall functionality of the micro/nanorobot swarm.

### 5.5 Multi-target potential field environment

**Fig. 6:**
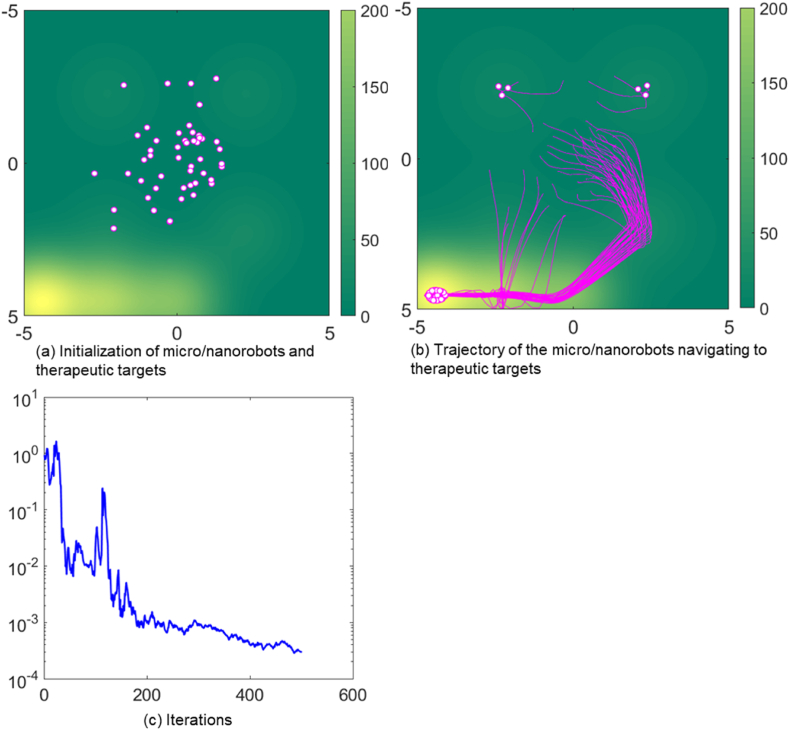
Trajectories of micro/nanorobots navigating to therapeutic targets in multi-target environments.

Fig. 6 shows the effect of the potential field peak associated with the therapeutic target on the collective motion of micro/nanorobots. In Fig. 6 (a), the therapeutic target with a potential field peak of 15 is clearly attractive to the micro/nanorobots, distinguished it from the other 5 regions with lower peaks. These peaks of the potential field correspond to the different levels of attraction of the drug and biological stimuli to the micro/nanorobots. The peaks of these potential fields symbolize different pharmacological or biochemical stimuli that exert different degrees of attraction on micro/nanorobots. Therefore, regions with a higher therapeutic effect and a greater need for targeted therapy are more attractive to micro/nanorobots due to their enhanced ability to sense biochemical signals. Fig. 6 (b) shows that micro/nanorobots are not directly attracted to the region with the highest peak potential for therapeutic effect. Instead, they continuously move towards the nearest therapeutic target and then gradually converge on the therapeutic target with the largest peak. This reflects that micro/nanorobots consider a combination of factors such as target proximity, value of therapeutic effect, and accessibility when processing biochemical signals and making decisions. Thus, micro/nanorobots initially engage with therapeutic targets that are closer and more directly accessible, before identifying and navigating towards the one with the highest peak.

As shown in Fig. 6 (c), the initial MSE value is high, which indicates that the distance between the current position of the micro/nanorobots and its therapeutic target is still far away. However, MSE values decrease rapidly as these micro/nanorobots move toward the nearest therapeutic target and learn from the biomedical environment in which they are located and their adjacent micro/nanorobots. This rapid decline demonstrates the effective progress of the micro/nanorobots toward their therapeutic goals. Moreover, due to the complexity of the human body and the presence of other therapeutic targets, the path to the therapeutic goal is rarely straightforward for these micro/nanorobots. This complexity leads to fluctuations in MSE values, which reflect the challenges encountered by micro/nanorobots in their navigation, learning process, and strategy adjustment. By continuously adapting and adjusting their behavior over iteration time, the MSE values showed a gradual decline and a trend toward stabilization, indicating that they took a consistent and collective approach to effectively achieve the therapeutic goal.

### 5.6 Extreme value environments

**Fig. 7:**
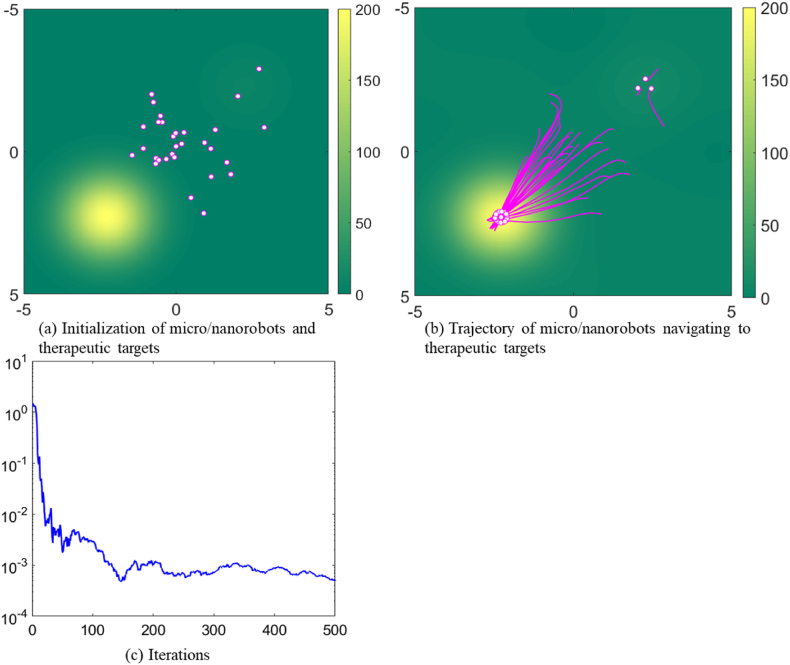
Trajectories of micro/nanorobots navigating to therapeutic targets in extreme value environments.

In Fig. 7 (a), thirty micro/nanorobots are randomly distributed within the environment, and the number of potential fields is set to two. The potential fields are depicted using a color gradient, where intensities in the yellow regions indicate higher potential values. The maximum potential value is 20 in the bottom left corner, while the minimum potential value is 1 in the top right corner. In Fig. 7 (b), the response of the micro/nanorobots to the potential fields is illustrated. The maximum potential field value of 20 in the bottom left corner exerts a strong attractive force, effectively drawing most of the micro/nanorobots towards this region. The motion paths of these micro/nanorobots are shown as pink traces, indicating their trajectories. The significant convergence of the micro/nanorobots towards the lower left region of high potential highlights their ability to navigate effectively towards areas of higher potential value. On the contrary, the minimum potential values close to 0 in the upper right corner exert much less influence, resulting in fewer micro/nanorobots navigating in that direction. This behavior demonstrates the micro/nanorobots' ability to respond to multiple targets within the environment, highlighting their flexibility and adaptability. The capacity to prioritize different targets based on the potential field values allows the micro/nanorobots to address multiple therapeutic needs simultaneously. This selective movement is crucial for minimizing unnecessary interactions and potential side effects in non-targeted regions, thereby enhancing the overall safety and effectiveness of the therapy.

As shown in Fig. 7 (c), in the initial phase, spanning from 0 to 50 iterations, the variable starts at a high value and experiences a rapid decrease within the first few iterations. This sharp decline indicates that the system quickly reduces the initial error or cost, making substantial progress toward the optimal solution. This phase is crucial for quickly reducing the initial positional errors and moving the micro/nanorobots towards areas of higher potential. The sharp decline in the convergence graph mirrors the micro/nanorobots' swift acceleration observed in the velocity plot, indicating effective initial convergence. By around the 50th iteration, the rate of decrease slows down, marking the transition to the next phase. The intermediate phase, covering iterations 50 to 200, is characterized by a slower rate of decrease and noticeable fluctuations in the variable's value. These oscillations suggest some instability or adjustments within the system, possibly due to the complexity of the optimization landscape or parameter tuning. In the later phase, from 200 to 500 iterations, the graph shows a more stable and gradual decrease in the variable's value. Minor fluctuations are still present, but the value steadily declines, suggesting that the system is fine-tuning the solution. By the end of the 500 iterations, the value stabilizes, indicating that the system has approached or reached an optimal state. This behavior demonstrates the robustness and adaptability of micro/nanorobots in achieving optimized navigation for targeted therapeutic interventions. The ability to quickly adjust, dynamically navigate, and stabilize movements ensures high precision in reaching therapeutic targets, optimizing the efficacy of drug delivery or other treatments.

**Fig. 8:**
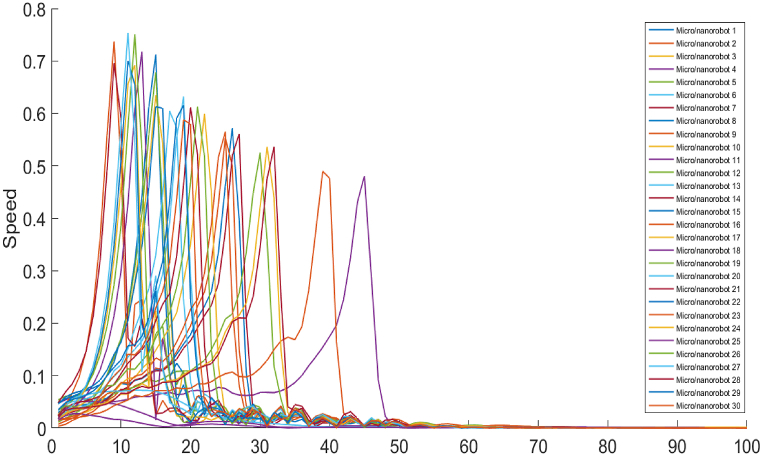
Speeds of micro/nanorobots over iterations.

Fig. 8 illustrates the convergence of the velocity for thirty micro/nanorobots over time. The x-axis represents time or iteration steps, while the y-axis shows the speed of each micro/nanorobot. During the initial phase, from 0 to 10 iterations, the velocities of the micro/nanorobots start from very low values and rapidly increase. This sharp rise indicates that the micro/nanorobots are quickly accelerating as they begin to respond to the potential fields. This initial acceleration corresponds to the rapid initial convergence observed in the previous convergence graph, where the micro/nanorobots quickly reduce the initial error or cost by moving towards regions of higher potential. In the intermediate phase, spanning from 10 to 40 iterations, the velocities of the micro/nanorobots exhibit significant fluctuations. These oscillations reflect the micro/nanorobots' interactions with the potential field and the adjustments they make to navigate effectively. The presence of these fluctuations is consistent with the oscillations observed in the intermediate phase of the previous convergence graph. In the later phase, from 40 to 100 iterations, the velocities of the micro/nanorobots stabilize and gradually decrease. This indicates that the micro/nanorobots are fine-tuning their movements, achieving a more stable and efficient navigation towards the target areas with high potential values. Overall, the ability to quickly accelerate, adjust to complex environments, and stabilize movements demonstrates the micro/nanorobots' robustness and adaptability in optimizing navigation for targeted therapy and drug delivery (see Fig. 8).

**Fig. 9:**
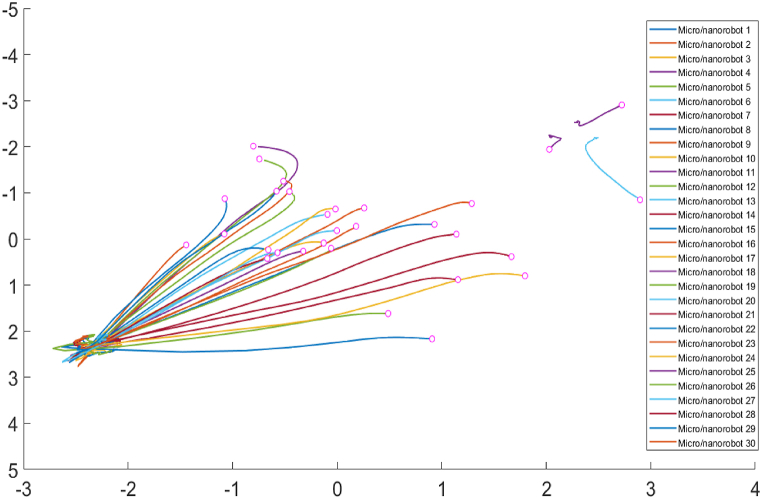
Micro/nanorobot trajectories from initial positions to final aggregation.

Fig. 9 depicts the paths of thirty micro/nanorobots from their initial positions to their final aggregation points. Each line represents the trajectory of an individual micro/nanorobot, color-coded for distinction, with pink dots marking the initial positions. It provides a dynamic visualization of how the micro/nanorobots navigate through the biomedical environment in response to the potential fields, complementing the insights gained from the convergence and velocity plots. In the initial phase, the micro/nanorobots are dispersed around their starting points. As the micro/nanorobots begin to gather in the potential fields, their trajectories exhibit significant fluctuations and adjustments, particularly evident in the intermediate phase. The fluctuations in velocity and the intermediate oscillations in the convergence graph reflect the dynamic interactions the micro/nanorobots have with the potential fields. During this phase, the micro/nanorobots encounter various obstacles or gradients in potential values, necessitating continuous adjustments to their paths. The trajectory plot shows that while most micro/nanorobots follow relatively smooth paths towards the bottom left corner (the high-potential region), a few micro/nanorobots deviate and navigate towards another potential peak in the upper right corner. This behavior illustrates the micro/nanorobots' ability to respond to multiple targets within the environment, showcasing their flexibility and adaptability in navigating complex terrains. In the later phase, the micro/nanorobots' movements stabilize as they approach their final aggregation points. The trajectories in the plot converge towards the target areas, with the majority of the micro/nanorobots aggregating in the bottom left corner, where the potential field is strongest. In conclusion, this precise and stable movement ensures that the micro/nanorobots can effectively concentrate their therapeutic actions in regions with higher potential values, optimizing the efficacy of targeted therapy and drug delivery.

## 6 Conclusion

In this study, we explored the integration of potential field mechanisms and collective behavior within micro/nanorobot swarms for enhancing targeted therapy and drug delivery. By simulating natural collective behavior, we aimed to overcome limitations associated with single micro/nanorobots, such as restricted loading capacity and navigational challenges in complex biomedical environments. Firstly, the results suggest that collective behavior can be used to improve the navigation, efficiency, and therapeutic effectiveness of micro/nanorobots. Through distributed learning and cooperative control strategies, swarm micro/nanorobots adjust their navigation in real-time, optimizing therapeutic results while minimizing off-target effects. Secondly, potential field mechanisms and collective intelligence enable microrobots to dynamically respond to environmental gradients, effectively target therapeutic sites with precision. This approach not only addresses the challenges of drug loading and delivery but also enhances the robustness of targeted therapy through redundant and distributed learning. The simulation results highlight the potential of this approach in achieving higher precision and efficiency in drug delivery, particularly in dynamically changing or challenging environments. Furthermore, this study emphasizes the importance of local interactions and collective decision-making in navigating complex environments. By simulating different scenarios, including standard, noise, density, communication-limited, and multi-target environments, we provided insights into the capabilities and adaptability of micro/nanorobot under various conditions. The observed reduction in the mean square error and efficient navigation to the therapeutic target in these simulations both demonstrate the effectiveness of our proposed framework. The ordinary differential equations method is used to analyze the convergence and stability of these algorithms, which ensures that the system remains robust and effective under changing conditions.

In summary, the research expands the interdisciplinary field of micro/nano robotics through simulating the advantages of collective behavior for targeted therapy and drug delivery. By integrating potential field mechanisms with distributed learning and cooperative control, we offer a strategy to overcome the intrinsic limitations of single micro/nanorobot. This study not only deepens our understanding of micro/nanorobotics in biomedical applications but also opens new possibilities for the development of more efficient, precise, and robust therapeutic delivery systems.

## Limitations and future work

Static noise can effectively simulate certain environmental variabilities and uncertainties, such as sensor inaccuracies, minor manufacturing differences, and consistent background interference. However, static noise does not represent all dynamic environments and has some significant limitations. These dynamic factors include fluctuating fluid flows, variable magnetic fields, and moving obstacles, all of which can significantly impact the navigation and functionality of micro/nanorobots. Our future research will focus on several key areas to overcome these limitations. First, we will incorporate dynamic noise models that better reflect the unpredictable and time-varying nature of real-world environments. Second, we will design realistic scenario-based tests that simulate specific biomedical environments. These tests will include navigating through the human circulatory system, moving within the gastrointestinal tract, or operating in tissue-like media. By tailoring the simulations to these scenarios, we can better evaluate the practical applications and limitations of our micro/nanorobots. Third, we will take interdisciplinary approaches, collaborating with experts in fluid dynamics, control theory, and biomedical engineering to enhance the realism and applicability of our simulations. Finally, we will validate our findings through experimental testing. By comparing the results of our dynamic simulations with real-world experiments, we can refine our models and control strategies, ensuring they accurately represent the complexities of biomedical environments. These efforts will ensure that micro/nanorobots are better prepared to navigate and operate effectively in complex biomedical environments, ultimately improving their potential for clinical applications.

## Data availability statement

The data that support this study are available from the corresponding author upon reasonable request.

## Ethics declarations

Review and/or approval by an ethics committee was not needed for this study because it donot include any human or animal participation.

## Funding

This work was supported by the 10.13039/501100001809National Natural Science Foundation of China (Grant Nos. 71571091, 71771112)

## CRediT authorship contribution statement

**Junqiao Zhang:** Writing – original draft, Visualization, Methodology. **Qiang Qu:** Visualization, Resources, Investigation. **Xuebo Chen:** Writing – review & editing, Methodology, Conceptualization.

## Declaration of competing interest

The authors declare that they have no known competing financial interests or personal relationships that could have appeared to influence the work reported in this paper.
